# Solar disinfection: an approach for low-cost household water treatment technology in Southwestern Ethiopia

**DOI:** 10.1186/2052-336X-12-25

**Published:** 2014-01-10

**Authors:** Awrajaw Dessie, Esayas Alemayehu, Seblework Mekonen, Worku Legesse, Helmut Kloos, Argaw Ambelu

**Affiliations:** 1 Department of Public Health, College of Health Science, Mekelle University, Mekelle, Ethiopia; 2 Department of Environmental Health Science and Technology, College of Public Health and Medical Sciences, Jimma University, Jimma, Ethiopia; 3 TREE Foundation, Texas, USA; 4 Department of Epidemiology and Biostatistics, University of California, San Francisco, California, USA

**Keywords:** Safe water supply, Water disinfection, Household water treatment, Solar radiation

## Abstract

Disinfection of contaminated water using solar radiation (SODIS) is known to inactivate bacteria. Its inactivation efficiency depends on local conditions where the disinfection is made. This study was aiming to test the efficiency of solar disinfection using different water parameters as low-cost household water treatment technology. Inactivation of microbes was tested using fecal coliform as test organism. The SODIS experiment was carried out at turbidity 2NTU, pH 7, and various water temperature (38.1°C, 41.8°C, 45.6°Cand 51.1°C) and solar intensities, using clear and black plastic bottles filled to different depths. The results show that the rate of microbial inactivation in relation to depth of water, turbidity, container type, intensity of light and color of container was statistically significant (p < 0.05). However, bottle placement, exposure and water pH were unrelated to microbial inactivation. Bacterial re-growth was not observed after solar disinfection. By adjusting the parameters, complete and irreversible fecal coliform inactivation was achieved within an exposure time of less than four hours in the areas where the solar irradiance is about 3.99 kW/m^2^ and above. Our results indicate that application of SODIS could play a significant role in the provision of safe water in rural communities of developing countries where there is ample sunshine, specifically in sub-Saharan African countries.

## Background

Ethiopia and some other developing countries have large fresh water resources. However, those countries cannot cover the expense of constructing water and wastewater treatment plants, distribution systems and the cost of the treatment processes for all residents. Globally, over 1.1 billion people are at risk of becoming infected with water-related pathogens due to lack of access to safe drinking water [1], a problem that is also widespread in Ethiopia [2].

Conventional water treatment plants in Ethiopia are scarce and the existing plants are vulnerable to frequent interruption and technical malfunction. Expanding treatment plants in rural areas is difficult due to logistics and scarcity of chemicals, energy, and lack of know-how [3]. Commercially produced filters are relatively costly, and filters made of locally available material are generally of limited treatment efficiency in improving microbiological water quality [4].

Several studies indicated that solar disinfection may be an alternative, low- cost, effective and simple method of water purification for use at the household level [5-7]. However, additional studies under different local environmental conditions (solar intensity, temperatures, precipitation), are necessary because the disinfection potential of SODIS varies from place to place. Adequate databases of environmental conditions in a given area of SODIS application may permit the development of models that could be used to test optimum disinfection potential [5]. No database exists on environmental parameters in Ethiopia to carry out SODIS experiments using coliform or other water-related pathogens.

## Methods

### Laboratory tests

A laboratory based experimental study was conducted in Jimma town, Ethiopia from April to June 7, 2011. Synthetic water deliberately contaminated with fecal matter was used as raw water.

Subsequently the efficiency of solar disinfection was evaluated using well water. The level of microbial inactivation was determined in the form of log inactivation following the method described below:

Log inactivation = Log10N_t_/N_0_

Where, N_t_ = count of microbes in CFU/mL at a time “t”.

N_0_ = initial count of microbes in CFU/mL

First order inactivation rate constant (k) of fecal coliform was computed based on Chick’s law [8] by performing linear regression analysis on plots of Ln (N/N_0_) versus “t”.

### Testing the efficiency of SODIS under different parameters

The efficiency of SODIS was tested under different levels of turbidity, water depths in bottles, water temperatures, and solar intensities. To determine the effect of raw water turbidity, raw water samples having turbidity levels of, 2, 13, 25, 46 and 81NTU were used. To determine the effect of water depth on SODIS, bottles having 0.5 L of 5.5 cm water depth, 1.0 L of 7.5 cm water depth, 1.5 L of 8.5 cm water depth and 2.0 L of 10 cm water depth were filled with raw water having initial bacterial load of 810 CFU/mL and exposed under direct sunlight. The effect of water temperature on SODIS efficiency was determined by exposing bottles on different surfaces (concrete, corrugated iron sheet (CIS) and cardboard) and by using half-surfaced black colored PET bottles and exposing them on a concrete surface.

The effect of solar intensity on SODIS was determined by exposing the bottles on three days with sky entirely covered with clouds, with few clouds and on fully sunny days, when solar radiance was recorded as 0.602 kWh/m^2^, 2.77 kWh/m^2^ & 3.99 kWh/m^2^, respectively.

SODIS bacterial inactivation efficiency was tested by adjusting turbidity of the water, water depth, solar intensity, and exposure time. This helps to identify optimum SODIS efficiency levels for direct applicability of the technology. The application of SODIS for naturally contaminated sources was further demonstrated by abstracting well water having turbidity of 13.0NTU and dissolved oxygen concentration of 3.24 mg/L. By aerating the water sample, the DO was increased from 3.24 mg/L to 4.86 mg/L. Possibility of microbial re-growth was determined by storing the treated water under room temperature for about four days.

### Analytical methods

#### Physical analysis

Physical parameters of water were measured following standard procedures [9] (see Table 1). Solar irradiance was calculated based on the equation: É*ϵ*As = Qconv + Qrad, where É = solar irradiance; ϵ = emissivity; As = surface area in contact with air; Qconv = rate of natural convection heat transfer; and Qrad = rate of radiation heat transfer [10].

**Table 1:** Physicochemical parameters and the instruments used for measuring, June, 2011, Jimma, Ethiopia

**Parameters**	**Measuring instrument**
pH	Portable Wagtech® 911 pH meter
Turbidity	Portable Wagtech® turbidity meter
DO	Multi-parameter probe HACH®
Exposure time	Stopwatch
Water temperature	Handheld Knick Portamess® 911 thermometer
Sunlight intensity (irradiance)	Non-contact thermometer and black body using mathematical equation

®: registered trademark symbol.

#### Microbial analysis

Samples were taken by shaking the exposed bottles to distribute fecal coliforms evenly in the bottled water. During sampling and transportation samples were protected from direct sunlight and transported in cold box at −8°C following standard methods [11]. The analysis was carried out within 6 hours of sample collection. Plate count agar was used for medium of bacterial growth and the sample was incubated at 44.5°C for 48 hours. Fecal coliform enumeration was done by a standard pour plate method [9]. This method was used so as to not getting running out of the water sample exposed for sunlight in frequent sampling by its advantage of using small water sample.

### Data analysis

Data were analyzed using SPSS Software for Windows Version 16. Descriptive statistics were performed to summarize the data in the form of mean and standard deviation. Non-parametric statistical tests were performed to test mean difference of log inactivation of fecal coliform and rate of inactivation among different variables at confidence level of (α-value = 0.05).

## Results

### Effect of raw water turbidity on SODIS efficiency

**Figure 1:**
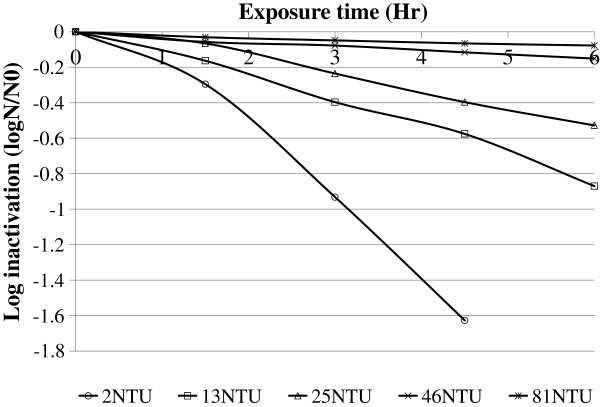
Log inactivation of fecal coliform for different turbidity level of water samples versus exposure time, June, 2011, Jimma, Ethiopia.

The highest log inactivation (0.93 ± 0.08) was obtained at turbidity value 2NTU. On the contrary, the least log inactivation (0.05 ± 0.005) was found at turbidity 81NTU (Figure 1). Log inactivation results were statistically significant between the different water samples having different turbidity (p = 0.009).

### Effect of water depth on efficiency of SODIS

**Figure 2:**
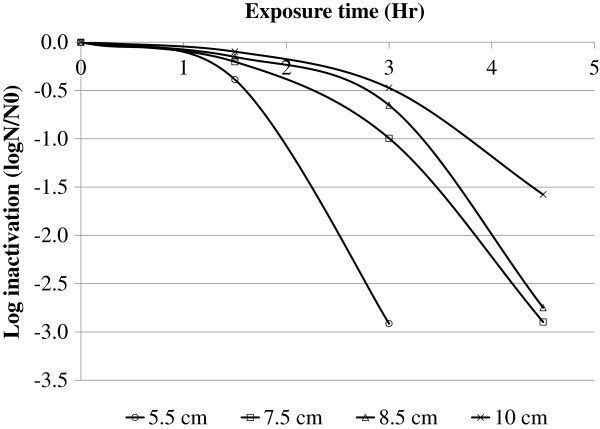
Log inactivation of fecal coliform on water samples having different water depth (5.5 cm, 7.5 cm, 8.5 cm and 10 cm), 2011, Jimma, Ethiopia.

Highest log inactivation of fecal coliform (2.91 ± 0.001) was found on water samples having water depth of 5.5 cm (Figure 2). The lowest log inactivation (0.474 ± 0.044) was obtained on 10 cm water depth after 3 hours of exposure. Kruskal-Wallis test on log inactivation of fecal coliform showed statistically significant differences among water samples with different water depths (p = 0.015).

### Effect of water temperature on SODIS

**Figure 3:**
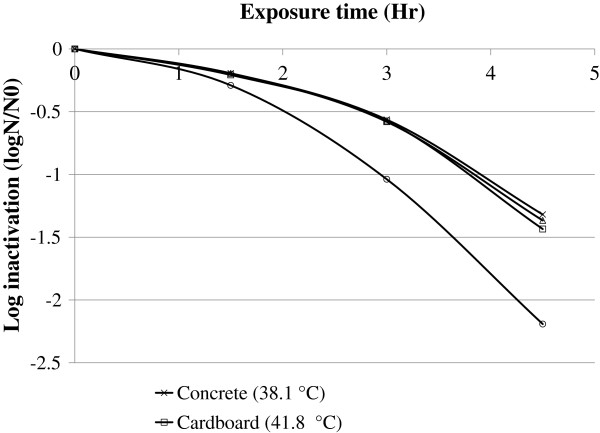
Log inactivation of fecal coliform on water samples exposed for sunlight on three different surfaces and under half-surfaced black colored PET bottles, June, 2011, Jimma, Ethiopia.

A significantly higher rate of bacterial inactivation was found using half-surfaced black colored PET bottle in comparison to raw water samples exposed on the surface of cardboard, concrete and CIS (Figure 3).

### Effect of solar intensity on SODIS

Within three hours of exposure, the highest log inactivation of fecal coliform (1.65 ± 0.05 log) was found on water samples exposed for sunlight having a cumulative solar irradiance of 3.99kWh/_m_^2^ and the lowest log inactivation (0.2 ± 0.01 log) and (0.95 ± 0.03 log) at cumulative solar irradiance of 0.6026kWh/m^2^ and 2.77kWh/m^2^. The mean log inactivation of fecal coliform found on the three different days was significantly different (Kruskal-Wallis test, p = 0.027).

### Testing the efficiency SODIS under adjusted parameters

**Figure 4:**
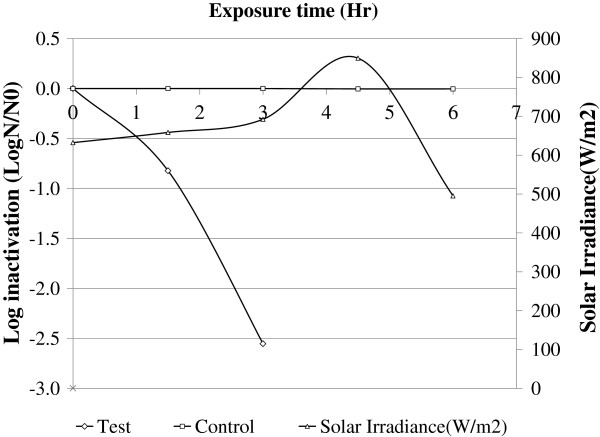
Log inactivation of fecal coliform on water samples exposed for sunlight under optimized conditions, June, 2011, Jimma, Ethiopia.

After the parameters were adjusted as described in the Methods section, SODIS efficiency was 2.554 ± 0.093 log inactivation after three hours of exposure under turbidity 2NTU, pH 7, DO of 6.52 mg/L, half-surfaced black colored PET bottle and water depth of 10 cm. No microbial inactivation was found in the control water samples (Figure 4). Mann–Whitney rank sum test on log inactivation of fecal coliform showed a statistically significant difference between the test and control water samples (p = 0.037).

**Figure 5:**
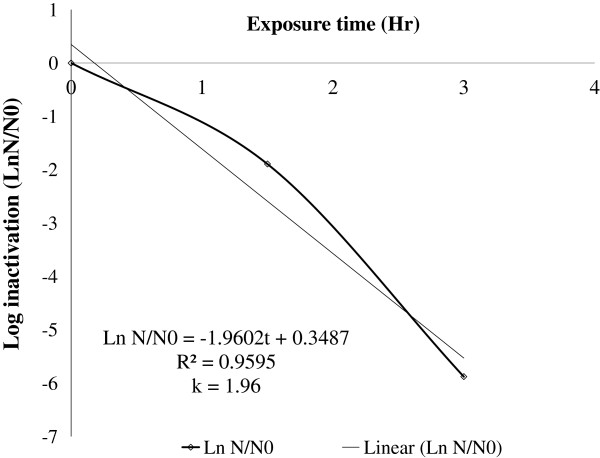
Inactivation rate constant (k) of fecal coliform contaminating synthetic raw water kept under half-surfaced black colored PET bottle, June, 2011, Jimma, Ethiopia.

The rate of fecal coliform inactivation was found to be (1.96 ± 0.071) per hour (Figure 5). The time taken to inactivate 99.9% of microbes (t_99.9_) was computed and showed that 3-log inactivation was achieved after (3.7 ± 0.12) hours exposure. The microbial re-growth test results showed that inactivated coliform bacteria failed to re-grow at ordinary room conditions after four days of storage in a dark place, or indicating that the inactivation process was irreversible.

### Application of SODIS for naturally contaminated water

**Figure 6:**
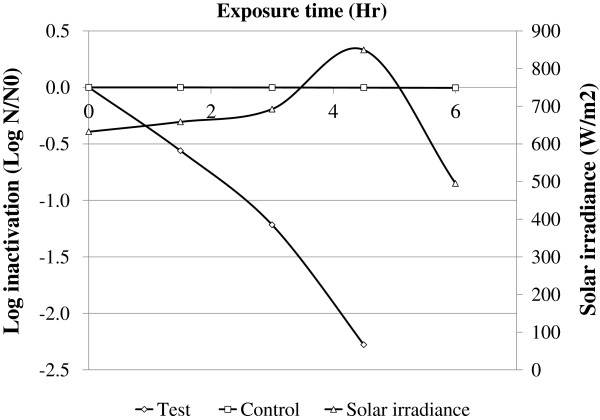
Log inactivation of fecal coliform on natural unprotected raw water samples kept under half-surfaced black colored PET bottle, June, 2011, Jimma, Ethiopia.

**Figure 7:**
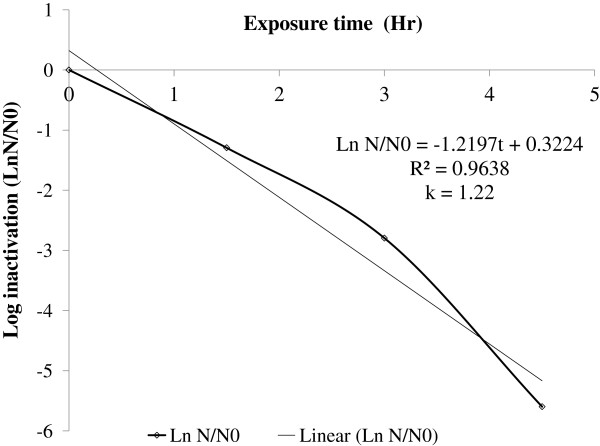
Inactivation rate constant (k) of fecal coliform contaminating natural (well) raw water, June, 2011, Jimma, Ethiopia.

The efficiency of SODIS was tested using naturally contaminated well water. The results show that within three hours of solar exposure, 1.22 ± 0.074 log inactivation of fecal coliforms was obtained and that no fecal coliform inactivation was observed in the control water samples (Figure 6). These differences were statistically significant (p = 0.046). The rate of microbial inactivation was found to be (1.22 ± 0.004) per hour (Figure 7).

## Discussion

The present study demonstrated that the applicability of low-cost household water treatment technology, using SODIS as an alternative method in the local setting. The results show log inactivation by sunlight was higher in clear raw water (< 5NTU) and that microbial inactivation was not significantly reduced water having turbidity values greater than 20 NTU. This might be due to shielding organisms by particles [12-14] and the reduction of the amount of UV radiation that penetrates the water [15]. This finding is in agreement with Wagelin et al. (1994) [16] emphasized that turbidity levels of raw water greater than 25NTU significantly reduce the disinfection efficiency of solar disinfection [16]. Therefore, if the turbidity of water is greater than 20NTU, the water needs to be pretreated before being exposed. Bigger particles and solids can be eliminated by storing the raw water for one day and letting the particles settle to the bottom [17]. Turbidity can also be reduced by flocculation/sedimentation using aluminium sulphate or crushed *Moringa oleifera* seeds, which are locally available in Ethiopia and other African countries [18].

This study demonstrated that higher bacterial log inactivation was found on water samples having shallow depth (5.5 cm) compared to water samples having 10 cm depth. This might be due to the reduction of intensity of UV radiation with increasing water depth [19]. Wagelin et al. (1994) [16] reported that at a water depth of 10 cm and moderate turbidity level of 26 NTU, UV-A radiation is reduced to 50% [16]. Commercially produced bags made from a transparent and a black PET-sheet (SODIS-bags) with a larger area for sunlight exposure and water depth of less than 6 cm may be a possible solution [3].

Regarding the effect of water temperature on SODIS efficiency, bacterial inactivation was higher by a factor of 1.68 in half-surfaced black colored PET bottles than in raw water samples exposed on cardboard, concrete and CIS surfaces, This higher inactivation rate can be explained by the high 51.1°C ± 0.17°C temperature of water obtained on clear sunny days. At this level of temperature synergistic effect between thermal inactivation and optical inactivation is expected [13,16]. This study also shows that exposing water on concrete, cardboard and CIS surfaces did not significantly affect the rate of microbial inactivation. This might be due to the fact that at the observed water temperature range (38°C to 45°C), the thermal effect on bacterial inactivation is limited [13].

The present study also demonstrates complete inactivation of *E. coli* within four hours of exposure using adjusted parameters for water disinfection. The system was also found to be effective in treating natural well water. The results indicate that complete microbial inactivation within four hours of exposure time could be convenient to parts of Ethiopia with a similar climate as Jimma town, including Nazaret, Mekele, Dire Dawa, which are receiving an average of 6.00, 5.54 and 5.96 kWh/m2 solar irradiance, respectively. The microbial re-growth test revealed that inactivated coliform bacteria fail to re-grow at ordinary room conditions after four days of storage in a dark place, indicating that the inactivation process was irreversible. Berney *et al*. (2006) [20] has got similar result with this finding on 5 days post treatment re-growth test [20]. Irreversible inactivation is highly important in relation to storing drinking water.

The potential benefits of SODIS technology in rural areas where safe water supplies are scarce are great. Rural communities relying on boiling as a disinfectant, typically use one kilogram of wood per liter of water, which is beyond the means of most households in sub-Saharan Africa [21]. By applying SODIS technology, a household can conserve 3,650 kg of wood per year, thus contributing to reduce degradation of the forests and bushes. Another method of rendering water safe for drinking, the use of water purification tablets, is even less affordable. One tablet of Aquatab®, which is supposed to treat 20 liters of water, is sold for about 0.633 US dollars. One household can save $US 116 (2204 Ethiopian Birr) per year using SODIS technology instead.

## Conclusions

In conclusion, the study has revealed the possibility of disinfecting microbial contaminated water with low- or no- cost technology in Jimma town in Ethiopia. The approach may be easily adapted to Ethiopian and other sub-Saharan communities with adequate sunshine which lack potable community water supplies as a strategy to reduce water-related diseases. For wider application of SODIS in various parts of developing countries, we recommend that further studies be carried out to determine seasonal variations in the efficiency and community acceptance of this technology.

## Competing interests

The authors declare that they have no competing interests.

## Authors’ contributions

AD, AA and EA designed the study, analyzed the data, drafted the manuscript and critically reviewed the article. SM, WL, and HK drafted the manuscript and critically reviewed the article. All authors read and approved the final manuscript.
